# Entropy of the resting state cortex in epilepsy

**DOI:** 10.1088/2057-1976/aea858

**Published:** 2026-10-06

**Authors:** Kirandeep Kaur, Jack O’Brien-Cairney, Gaurav Singh, Manoj Upadhya, Aphrodite Chakraborty, Sarat P Chandra, Harald Prüss, Hans-Christian Kornau, Dietmar Schmitz, Gavin Woodhall, Richard Rosch, Maia Angelova, Stefano Seri, Manjari Tripathi, Sukhvir K Wright, Caroline Witton

**Affiliations:** 1 Institute of Health and Neurodevelopment, College of Health and Life Sciences, Aston University, Birmingham, United Kingdom; 2 Wellcome Centre for Human Neuroimaging, University College London, London, United Kingdom; 3 MEG Resource Facility, National Brain Research Centre, Manesar, India; 4 Department of Neurology, All India Institute of Medical Sciences, New Delhi, India; 5 Department of Neurosurgery, All India Institute of Medical Sciences, New Delhi, India; 6 Department of Neurology and Experimental Neurology, Charité—Universitätsmedizin Berlin, Corporate member of Freie Universität Berlin and Humboldt-Universität zu Berlin, Berlin, Germany; 7 German Center for Neurodegenerative Diseases (DZNE) Berlin, Berlin, Germany; 8 Charité—Universitätsmedizin Berlin, corporate member of Freie Universität Berlin and Humboldt-Universität zu Berlin, Neuroscience Research Center, Berlin, Germany; 9 Department for Basic and Clinical Neuroscience, Institute of Psychiatry, Psychology and Neuroscience King’s College London, London, United Kingdom; 10 Aston Digital Futures Institute, College of Engineering and Physical Sciences, Aston University, Birmingham, United Kingdom; 11 Birmingham Women’s and Children’s Hospital NHS Trust, Birmingham, United Kingdom; 12 Humboldt-Universität zu Berlin, Bernstein Center for Computational Neuroscience, Berlin, Germany; 13 Department of Clinical Neurophysiology, Birmingham Women’s and Children’s NHS Foundation Trust, Birmingham, United Kingdom

**Keywords:** epilepsy, MEG, EEG, entropy

## Abstract

Epilepsy has long been conceptualized as a disorder in which aberrant brain dynamics extend beyond the epileptogenic zone. Evidence demonstrates that loss of entropy is a generic feature of pathological dynamics in the brain, including the ictal state. However, the impact of recurrent seizures on entropy in the interictal state remains unknown. Resting state magnetoencephalography (MEG) scans and resection masks of 32 individuals with epilepsy who had Engel I outcome post-surgery were retrospectively retrieved. Using co-registered FreeSurfer parcellations, we reconstructed the source localized MEG time series and computed sample entropy (SE) for 114 regions of interest. We then tested the association of entropy with the resected volume of the brain, and additional clinical variables including the age of seizure onset, seizure frequency and duration of epilepsy. To further understand the temporal relationship between seizure onset and entropy in the interictal state, we collected and computed SE for week-long electroencephalography traces from leucine-rich glioma inactivated 1 monoclonal antibody (LGI1-mAb) rodent models of autoimmune encephalitis (*n* = 5) and control rats (*n* = 5). We found that in individuals with epilepsy, a lower age of seizure onset was associated with lower mean SE of the whole cortex (Spearman’s rho = 0.60, *p* < 0.001; partial correlation = 0.41, *p* = 0.021). Entropy did not differ between the resected and non-resected regions of the brain. Furthermore, in an exploratory analysis, LGI1-mAb treated rodents showed lower SE as compared to control rats, after the onset of seizures. Together, the results indicate that cortical entropy may serve as a marker of long-term alterations in cortical dynamics associated with recurrent seizures.

## 1. Introduction

In nature, complex systems are constituted of a large number of interacting parts which collectively give rise to patterns of functioning and/or behaviour that cannot be explained by the laws governing the constituent parts themselves [1, 2]. For example, starling murmuration (an observable phenomenon where thousands of starling birds fly together in synchronized and continuously shifting patterns), cannot be explained by the behaviour of a single starling alone [3]. Likewise, the human brain is another example of a complex system where individual neurons collectively give rise to higher order functions like language and cognition [4, 5].

Many complex systems including the mammalian brain are hypothesized to function in a state of *criticality*, which is an intermediate state between complete order and randomness [6, 7] and where complexity is maximal [8]. It is posited that systems in a state of criticality have the greatest capacity for adaptation and information processing [9, 10]. However, loss of complexity in neuronal activity is a hallmark of natural ageing, altered states of consciousness as well as selected brain disorders [11–14].

Complexity of a system is challenging to quantify [15]. Entropy, which is fundamentally regarded as the degree of order or randomness is a system has been used to measure the complexity in natural systems [16] ^(p1)^. At a foundational level, complexity is considered to be an emergent property of the continuously increasing entropy in the universe [17]. There exist several interpretations of entropy in neural systems ranging from the degree of disorder in neural signals and the information processing capacity of the brain [12, 18, 19], to the number of varied states or configurations in which the brain as a neural network can exist [20–22].

In patients with epilepsy, brain entropy falls sharply during an ictal event because neural activity during seizures becomes more synchronous and predictable [23–25]. Evidence from animal models of epilepsy suggests that recurrent seizures can lead to temporary or even permanent functional changes in synaptic activity by changing its threshold [26–28]. Therefore, we hypothesized that given its propensity for recurring seizures, the entropy of the epileptogenic zone would be lower as compared to the non-epileptogenic brain regions. The regional differentiation may serve as an additional tool to localize the epileptogenic zone.

To test this hypothesis, we computed sample entropy (SE) from source localized resting state (interictal) magnetoencephalography (MEG) data of patients who had undergone epilepsy surgery and had Engel I outcome for at least three years post-surgery. SE is a robust measure of the degree of irregularity or complexity in a time-series signal [29, 30]. In addition, entropy derived from human data was tested for its association with other clinical variables such as age of seizure onset and duration of epilepsy. To further understand the temporal relationship between seizures and abnormal interictal brain dynamics, we evaluated how entropy changes with the onset of epileptogenesis by analysing electroencephalography (EEG) traces from leucine-rich glioma inactivated 1 monoclonal antibody (LGI1-mAb) rodent models of autoimmune encephalitis and control rats. LGI1-mAb rodents provide an established model of autoimmune epilepsy, in which patient derived LG1 monoclonal antibodies increase neuronal excitability and elicit spontaneous epileptic seizures [31]. In this study, the experimental model was used to assess any change in entropy, before and after the onset of seizures.

## 2. Methods

### 2.1. Acquisition and pre-processing of MEG data

All clinical data was collected retrospectively with approval from the institutional ethics committee of All India Institute of Medical Sciences, New Delhi, India (Ref No.: IEC-256/04.03.2022). The clinical history, and MEG data were retrieved for 36 patients who had Engel I outcome (complete seizure freedom) for a duration of at least three years following surgery. Retrieving data only from patients who had complete seizure freedom post-surgery reduces uncertainty regarding the source of epileptogenic activity.

The source-localized MEG data, parcellated structural MRI, and the resection masks used here were reanalysed from a previously reported cohort [32], and included 32 retrievable datasets. An overview of the methodology for MEG source analysis and creation of resection mask is given below. The resection masks were used as a clinically informed anatomical proxy for the epileptogenic zone, indicating the tissue whose removal was sufficient to achieve sustained seizure freedom.

T1-weighted MRIs were pre-processed in FreeSurfer [33],using the Lausanne parcellation scheme [34]. For this study, we conducted our analysis over 114 neocortical regions of interest (ROI), excluding any deep brain structures such as hippocampus, thalamus and amygdala. The following steps were used to create masks for the regions which were resected during surgery: (i) FSL FLIRT was used to linearly co-register the postoperative MRI/CT (displaying the resection cavity) and the preoperative Freesurfer parcellated MRI [35, 36]. In cases where only the post-operative CT was available, the ITK-SNAP tool was used to adjust the contrast [37], (ii) masks were manually drawn over the resection cavity in FSLView and smoothened using the *fslmaths* toolbox.

Resting-state MEG was acquired for all patients preoperatively in an eyes closed, resting-state, supine position, using a 306-channel Elekta Neuromag system (Helsinki). Patient data was monitored continuously during acquisition, and periods where patients entered sleep states were annotated and excluded from the current analysis. No sleep deprivation procedures were used. Interictal epileptiform discharges (IEDs) were not systematically removed or quantified for this analysis. MEG was recorded at 1 kHz sampling frequency and corrected for head movement artefacts using the method of temporal signal space separation [38]. Further details of MEG acquisition protocols for these patients have been described previously [39, 40]. Raw MEG data were down-sampled to 500 Hz for computational efficiency. All further pre-processing was done in Brainstorm software [41]. The MEG data were co-registered with the parcellated structural MRI and band-pass filtered between 1–100 Hz. It was further notch-filtered at 50 Hz using a second-order infinite impulse response filter. Signal space projection was used to remove eye movement and electrocardiographic artefacts [42, 43]. The next step was to generate a single MEG time series for each ROI across the whole cortex. This was done by constructing a head model from the structural MRI for every patient using the overlapping spheres method. The sLORETA algorithm [44] was used to create a source model of the MEG signal across 114 neocortical regions.

### 2.2. Patient characteristics

The clinical and demographic characteristics of 32 patients are provided in table 1. As mentioned previously, all patients had reported an Engel I outcome for at least 3 years post-surgery. Duration of epilepsy was defined as the interval between age of seizure onset and age at the time of MEG acquisition. In addition, the following clinical variables were retrieved for each patient: the presence of focal-to-bilateral tonic–clonic seizures, lobe(s) of resection, and the number of concurrent antiseizure medications (ASMs). Concurrent benzodiazepine and barbiturate use was also recorded because these medications can alter background electrophysiological activity and could therefore influence signal-derived measures [45, 46].

**Table 1.** Clinical and demographic characteristics of patients.

Clinical and demographic characteristics	
Age [Mean ± SD]	15.91 ± 8.86

Duration of epilepsy in years [Mean ± SD]	8.09 ± 6.35

Age at seizure onset in years [Median(IQR)]	5.75 (1.5–13.0)

Seizure frequency/week [Median (IQR)]	7.0 (1.44–19.25)

Sex • Female (%) • Male (%)	12 (37.5) 20 (62.5)

Lateralization of the region resected during surgery • Left (%) • Right (%)	14 (43.75) 18 (56.25)

### 2.3. Acquisition and pre-processing of rodent EEG

Male wistar rats, at postnatal age day 21 (P21) were used to gather EEG data and recorded for a period of 21 d (*N* = 10). All animal procedures were conducted in accordance with current UK Home Office requirements under the Animals (Scientific Procedures) Act 1986, under the authority of an approved UK Home Office project licence, and in accordance with ARRIVE guidelines. Local ethical approval was granted by the Aston University Bioethics Committee, Birmingham, UK.

The rats were infused with patient-derived anti-LGI1 monoclonal antibodies or control antibodies (non-brain reactive) intracerebroventricularly via two subcutaneously implanted osmotic pumps (Alzet model 1007D, volume 100 uL, flow rate 0.5 uL per hour, duration 7 d). Out of the 10 rats used, five were infused with control antibodies and five were infused with human derived monoclonal anti-LGI1 antibodies [31]. Paired video-EEG recordings were collected using a previously standardized protocol at a sampling frequency of 512 Hz [47, 48].

The rodent EEG on the other hand was divided into non-overlapping hour-long bins. The total duration of rodent EEG recordings totalled 192 h (∼8 d). Seizure frequency was quantified as the total number of seizure events occurring per rodent in each hour long bin. To construct an appropriate analogue to human interictal MEG data, ictal events within the rodent EEG were first identified using a standardized protocol [31, 49] and omitted from further analysis.

### 2.4. Computing entropy

We used SE to measure the entropy of MEG source localized time-series and rodent EEG. SE measures the degree to which a time-series has similar or self-repeating patterns of a given length [50]. Mathematically, SE is the negative natural logarithm of the estimated conditional probability that two sequences similar for *m* consecutive points remain similar when their length is extended to *m* + 1 points, excluding self-matches [29, 50]. The lowest possible value of SE is 0 (indicating a higher degree of regularity or self-repetition in a time-series), while greater values indicate greater irregularity or complexity. In noise free data, a lower value of entropy indicates recurrence or regularity of neural signal, and may reflect a more constrained or repetitive underlying neural dynamics.

SE is very similar in computation to another measure of regularity of a time-series, approximate entropy, except that it excludes self-matching of sequences [16, 51]. This makes SE a more robust tool for measuring the presence of stochasticity (randomness) or regularity in a time-series over shorter durations [50].

SE was computed for the first 9 min (540 s) of each of the 114 ROI time series per patient. This duration was concluded to be sufficient since the mean SE across patients was found to stabilize and remain approximately constant after 360 s (6 min) (efigure 1). Given a sampling frequency of 500 Hz, 9 min of data corresponded to 270 000 data points per ROI. In line with recommendations by Delgado-Bonal *et al* [29], the tolerance, ‘*r*,’ for accepting matches, i.e. the degree to which a sequence can deviate from another and still be considered similar, was set as 0.2 SD (20% of the standard deviation of the time series). The length of the sequences (*m*) to be compared, was set as three. To assess the robustness of the findings to SE parameter selection, sensitivity analyses were performed across nine combinations of embedding dimension ‘*m*’ (2, 3, and 4) and tolerance parameter ‘*r*’ (0.15, 0.20, and 0.25). Owing to computational constraints, these analyses were conducted using 240 s of MEG data rather than the complete 9 min recording. The 240 s duration was considered adequate because whole-cortex mean SE of all patients estimated from 240 s differed by <1% from that estimated using the complete 9 min recording (efigure 1). Entropy was computed from each neural time series signal in MATLAB 2021a using the function developed by Brian Lord.

Since changes in SE can partly reflect a shift in spectral power [52] as opposed to a shift in temporal regularity of the time series, we also tested the association of SE with relative spectral power in delta (1–3 Hz), theta (4–7 Hz), alpha (8–12 Hz) and beta (13–30 Hz) bands, broadband power (1–100 Hz) and mean signal variance.

As described above, the rodent EEG was epoched into one hour-long analysis bins, SE was computed for each of these epochs using the same parameters and function.

### 2.5. Statistical analysis

Statistical analysis was conducted in R (version 2023.06.2) and MATLAB_R2021a.

For human MEG data, non-parametric tests were used to test the difference in average SE between the hemispheres ipsilateral and contralateral to the site of resection, and the resected and non-resected regions of the brain. Stepwise backward multiple linear regression was used to examine associations between the whole-cortex mean SE and the clinical and demographic variables. Backward elimination was based on the Akaike information criterion (AIC), and the model having the lowest AIC was retained, thereby balancing model fit against model complexity. Given the relatively large number of candidate predictors in relation to the sample size, a leave-out-analysis was performed on the complete backward-selection procedure by omitting each patient by turn. The frequency with which each predictor was retained, together with the direction and range of its coefficient, was examined to assess whether variable selection was driven by a subset of patients.

As an additional sensitivity analysis, all candidate predictors were entered simultaneously into a gaussian elastic-net penalized regression implemented using the ‘glmnet’ package in R. Predictors were standardized before penalization. The elastic-net mixing parameter, *α* was evaluated across values ranging from a mixture of ridge and LASSO regression (*α* = 0.1, 0.25, 0.5, 0.75, 0.9) to pure LASSO regression (*α* = 1). For each value of *α* the penalty parameter, *λ*, was evaluated using leave-one-out cross-validation, with mean squared error used to quantify prediction error. The value of α associated with the lowest cross-validated mean squared error was selected. For the primary penalized model, *λ* was chosen using the one-standard-error rule to favour a more parsimonious model. Predictors with non-zero coefficients were considered retained by the penalized model.

In cases where a confounding variable was found to impact both the independent and dependent variables, partial correlation was used to adjust for the confounding variable. This was done by regressing out the confounding variable from the dependent and independent variables, and then testing the residuals of these models for the presence of any significant correlations.

To test if the association between clinical variables and cortical entropy could be explained by spectral and/or amplitude-related characteristics of the MEG signal, additional sensitivity analysis was performed using linear regression. Relative spectral power in bands, absolute broadband power (1–100 Hz), and mean signal variance were examined separately. Nested models were compared to determine whether inclusion of clinical variables explained additional variance in cortical entropy beyond each spectral or signal characteristic.

We fitted a linear mixed-effects models to examine whether SE trajectories differed between LGI1-mAb and control rodents across one week of recording period. Mixed-effects models accounts for repeated observations within individual rodents, thereby avoiding the assumption that all hourly measurements are independent. In this model group, recording hour, and their interaction were included as fixed effects, with a random intercept for each rodent. The group-by-hour interaction tested whether the rate of change in SE differed between LGI1-mAb and control rodents. Because measurements taken close together in time may remain correlated even after accounting for rodent-level differences and the linear time trend, a second model incorporated a first-order autoregressive [AR(1)] residual correlation structure. Model fit with and without the AR(1) structure was compared using the AIC. Both models were fit using restricted maximum likelihood.

Cross-correlation (CCF) function was used to find the lagged correlation between the mean seizure frequency and SE per hour. The ‘astsa’ package in R was used to detrend the time-series of seizure frequency and SE [53].

## 3. Results

### 3.1. Human MEG

#### 3.1.1. Entropy is highly variable between patients

The mean SE of the whole cortex across patients was 0.84 ± 0.12. Interestingly, the variability of mean SE between patients (SD = 0.12) was significantly greater than the variability of the mean SE between ROIs (SD = 0.04). This indicates that the entropy of the whole cortex differed majorly from patient to patient, but did not show as much variability between different regions of the same cortex.

Therefore, contrary to our hypothesis, we did not find any significant difference in the mean SE of the resected and non-resected regions of the brain. In addition, mean SE did not differ significantly between the hemisphere ipsilateral and contralateral to the site of resection. We further compared and found no significant difference between SE in the ROIs in the resection volume and their homologous counterparts in the contralateral hemisphere.

### 3.2. Age of seizure onset is a predictor of entropy

To test the degree to which clinical characteristics of patients can predict their average SE across the cortex, we used stepwise regression analysis in the backward direction till the lowest possible AIC score was reached. The lowest AIC model retained age of seizure onset, resection in the parietal lobe and the intake of barbiturates as ASMs as predictor variables. Thus, earlier seizure onset, resection in parietal lobe and concurrent barbiturate use was associated with lower mean SE across the whole cortex. These variables together explained 50% of the variance in SE (*R*^2^ = 0.5, adjusted *R*^2^ = 0.45, *F*_3,28_ = 9.34, *p* = 0.0002; table 2). The findings regarding use of parietal lobe resection and barbiturate use necessitate caution because only three patients underwent parietal lobe resection and five received barbiturates in the entire patient cohort.

**Table 2.** Predictor variables retained in backward step-wise regression model of whole cortex mean SE.

Predictor		Estimate	Standard error			95% CI	*t*	*p*
(Intercept)		0.782	0.023			[0.735, 0.829]	34.17	<0.001
Age of seizure onset		0.01	0.002			[0.006, 0.015]	4.99	<0.001
Parietal lobe resection		−0.115	0.055			[−0.226, −0.003]	−2.1	0.0449
Barbiturates		−0.096	0.044			[−0.185, −0.007]	−2.21	0.0352

*N*	*R^2^*	Adjusted *R^2^*		*F*	df1	df2	Model *p*-value	AIC

32	0.5	0.447		9.34	3	28	<0.001	−59.39

Age at seizure onset was retained in all 32 leave-one-patient-out models, with a consistently positive coefficient (range: 0.0088–0.0143). Parietal lobe resection and barbiturate use were retained in 31 and 30 models, respectively, and their coefficients remained negative throughout. The penalized regression model retained the age of seizure onset as the only predictor variable (coefficient = 0.002). Collectively, these sensitivity analyses identified age at seizure onset as the most robust predictor of whole-cortex mean SE (coefficient = 0.01, *p* < 0.001). Duration of epilepsy was excluded from regression because it is mathematically dependent on age at scan and age at onset.

A scatterplot between the age of onset and the average entropy across the cortex is shown in figure 1(A). In addition age of onset was found to be correlated not only with the variability of SE between patients, but also between regions of the cortex (range of SE) within a patient (rho = − 0.5, *p* = 0.004; figure 1(B)). The association between SE and the age of seizure onset remained significant even after adjusting for the patient’s age at the time of scan (rho = 0.41, *p* = 0.021; figure 2).

**Figure 1.**
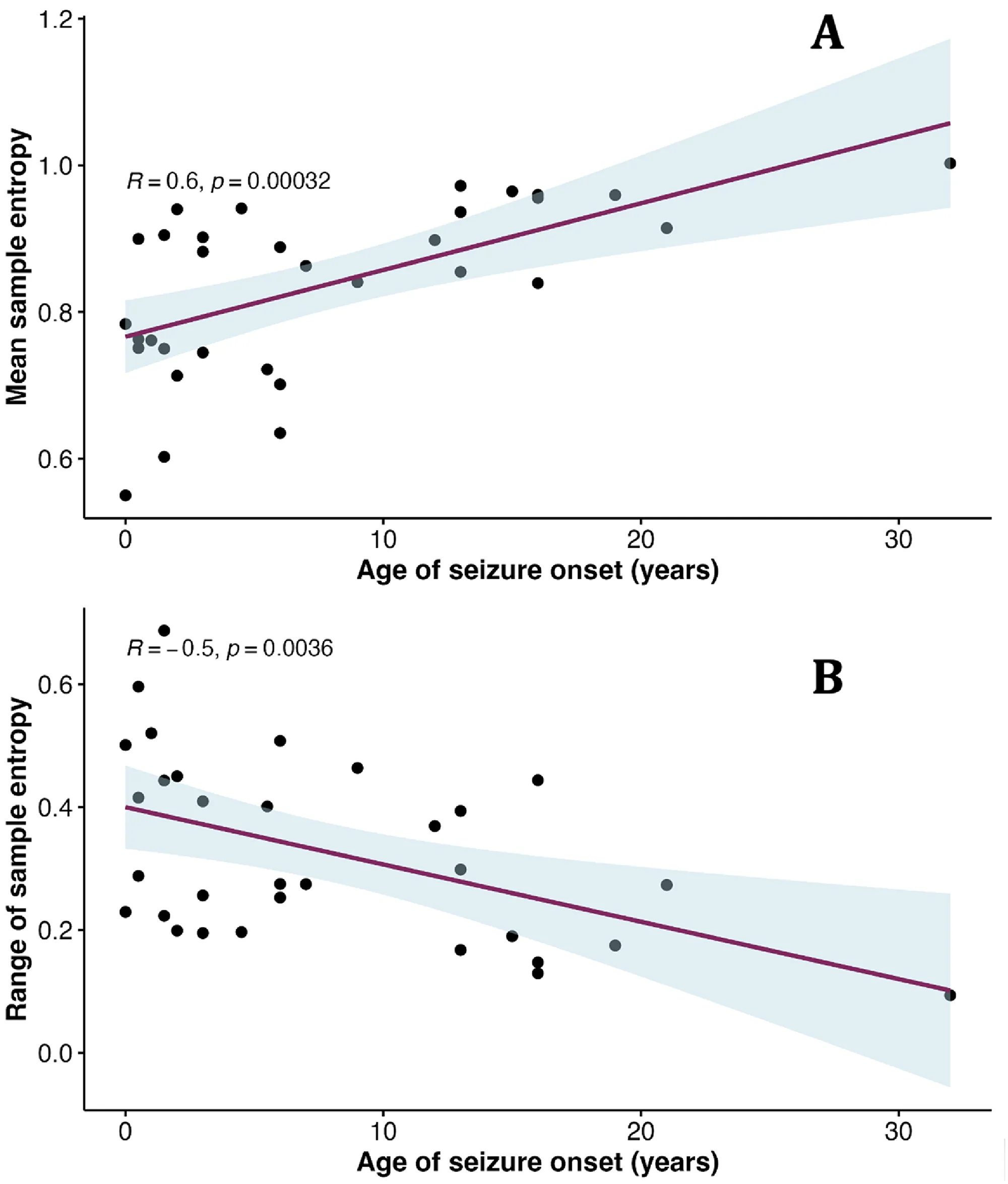
Age of seizure onset (years) correlates with the mean and range of sample entropy in the whole cortex. (A) The scatter plot shows the association between average SE across 114 ROI in each patient and their age of seizure onset in years. Each data point corresponds to one patient. The correlation coefficient (Spearman’s rho) was equal to 0.6 (*p* < 0.001). The maroon line depicts the regression fit. (B) Similarly the range (maximum-minimum) of entropy values within a patient was negatively correlated with their age of onset (Spearman’s rho = − 0.5, *p* = 0.004). This demonstrates that lower age of epilepsy onset is associated with greater variability of resting state SE between different regions of the cortex.

**Figure 2.**
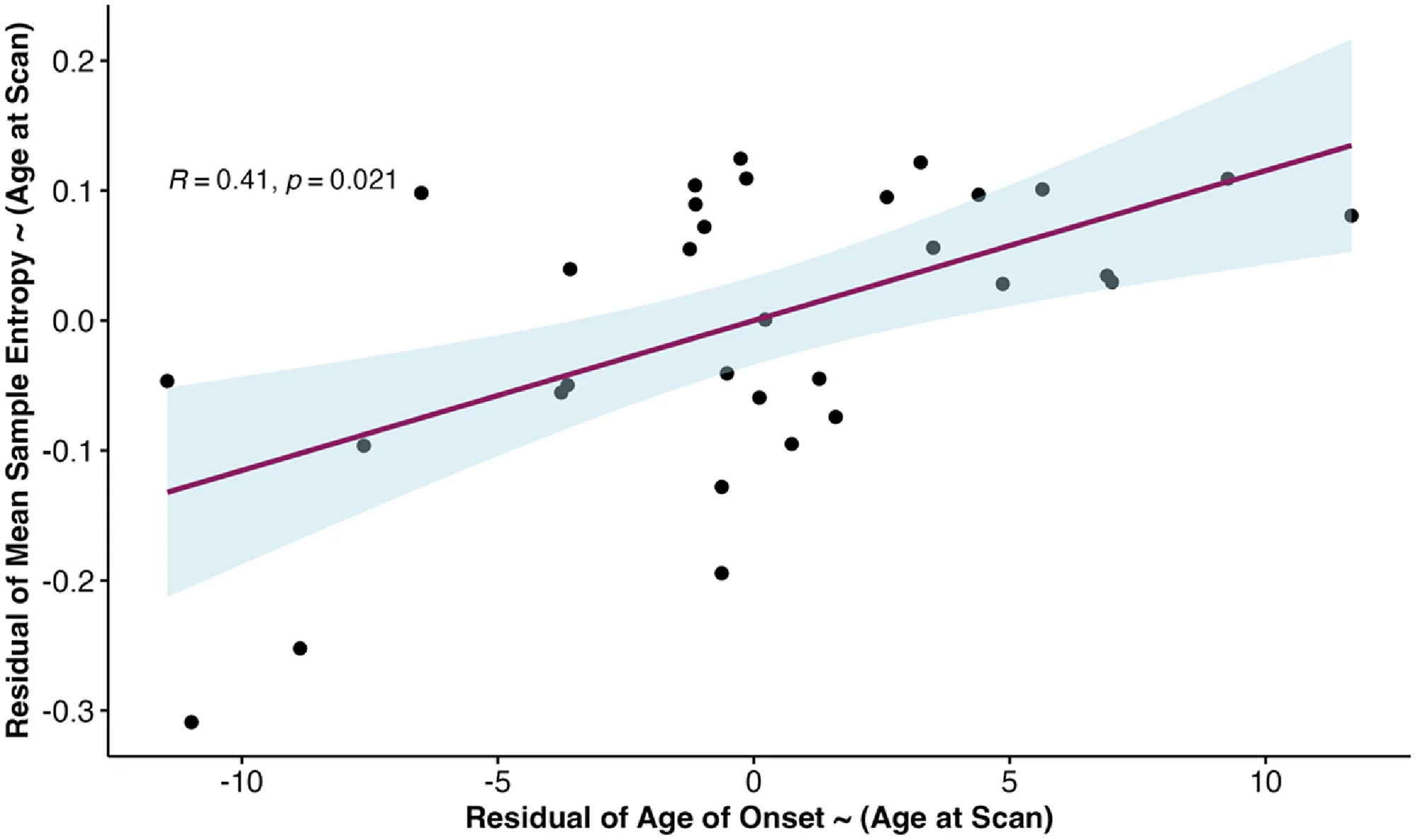
Partial correlation between age of seizure onset and mean sample entropy after adjusting for the age at scan. The scatter plot shows the correlation between the residuals of age of onset and the residuals of the mean SE after a regressing out the age at the time of MEG scan. The correlation coefficient (Spearman’s rho) was equal to 0.41 and remained statistically significant (*p* = 0.02).

Relative power in delta and theta frequency bands, broadband power and mean signal variance significantly predicted SE. However, the association between age of seizure onset and SE remained significant after adjusting for spectral power and signal variance. Addition of age of seizure onset significantly improved model fit beyond relative spectral power in bands (*F* = 5.52, *p* = 0.027), absolute broadband power (*F* = 10.34, *p* = 0.003), and mean signal variance (*F* = 11.46, *p* = 0.002).

Lastly, the association between SE and age at seizure onset remained robust across all tested combinations of tolerance (‘*r*’) and embedding dimension (‘*m*’) used to compute SE (eTable 1).

Together, these results indicate that an earlier age of onset of seizures is associated with lower entropy of the whole cortex, and this association is evident during the interictal state as well.

## 4. Rodent EEG

### 4.1. SE trajectory of LGI1-mAb infused rodent EEG differs from controls

We used a linear mixed effects model to test if SE measured over repeated hours differed between LGI1-mAb infused rats and controls. The analysis included 1682 h observations from 10 rodents. Incorporating an AR(1) residual correlation structure substantially improved model fit compared with the random-intercept model without autocorrelation (AIC: −1996.57 versus −1545.94). The estimated first-order autocorrelation was φ = 0.50 indicating moderate residual correlation between measurements recorded one hour apart.

At the beginning of recording, SE did not differ significantly between LGI1-mAb and control rodents (coefficient = − 0.039, *p* = 0.59; table 3). SE increased over time in control rodents (coefficient = 0.002 per hour, *p* < 0.001; table 3; figure 3), while the significant negative group-by-hour interaction indicated a substantially weaker increase in LGI1-mAb rodents compared to controls (coefficient = − 0.002 *p* < 0.001; table 3; figure 3). The estimated slope in the LGI1-mAb group was close to zero (coefficient = 0.0001), demonstrating progressive divergence in SE trajectories between the two groups (figure 3).

**Figure 3.**
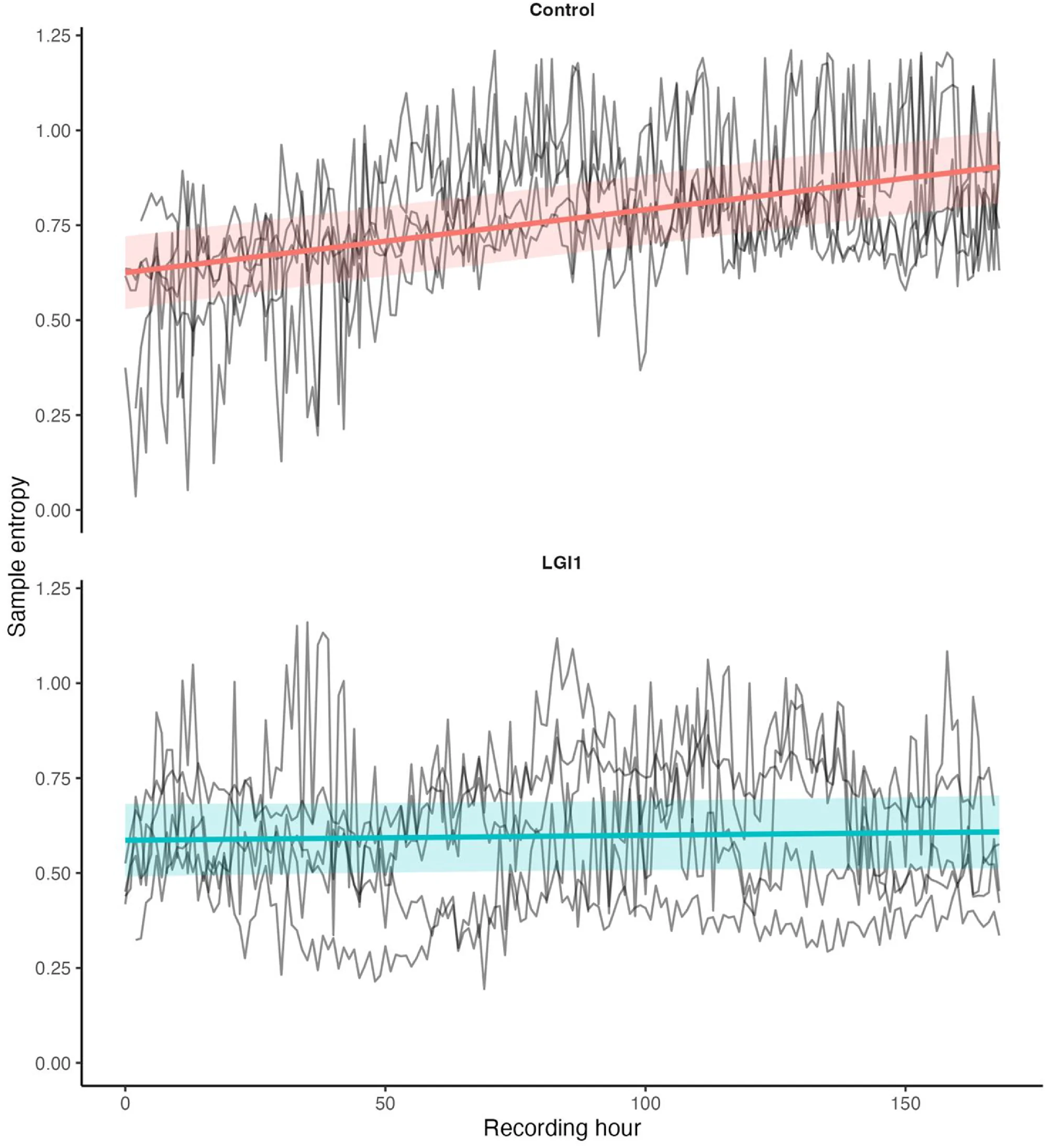
SE trajectories in LGI1-mAb infused and control rodents. The plot shows SE trajectories of individual rodents in LGI1-mAb and control group (n=5 per group). Bold coloured lines represent the group-level trajectories estimated from the linear mixed-effects model incorporating a random intercept for each rodent and an AR(1) residual correlation structure. Shaded regions indicate the 95% confidence intervals around the model-estimated trajectories.

**Table 3.** Linear mixed-effects model of SE trajectories in LGI1-mAb and control rodents.

Predictor	Estimate		Standard error	95% CI	*t*	*p*
Intercept	0.625		0.049	[0.529, 0.721]	12.79	<0.001
LGI1-mAb vs Control at hour 0	−0.039		0.069	[−0.198, 0.121]	−0.56	0.590
Recording hour: Control	0.002		<0.001	[0.001, 0.002]	9.13	<0.001
LGI1-mAb × recording hour	−0.002		<0.001	[−0.002, −0.001]	−5.95	<0.001

Observations	Rodents	AIC		BIC	Log-likelihood	AR(1) phi

1682	10	−1996.57		−1958.59	1005.28	0.5

### 4.2. Negative correlation between SE and seizure frequency in LGI1-mAb infused rodents

Further, we aimed to understand how the entropy of LGI1-mAb infused and control rats evolves in time, specifically after the onset of seizures. Given the small sample size for the LGI1-mAb infused rats (*n* = 5), the analysis in this section was considered exploratory in nature. We observed that SE trajectories showed significant divergence because SE increased in controls but remained comparatively stable in LGI1-mAb rodents after the onset of seizures (figure 4). Additionally, the difference in SE between the two groups showed a sustained difference even after cessation of the peak seizure frequency (table 3, figures 3 and 4).

**Figure 4.**
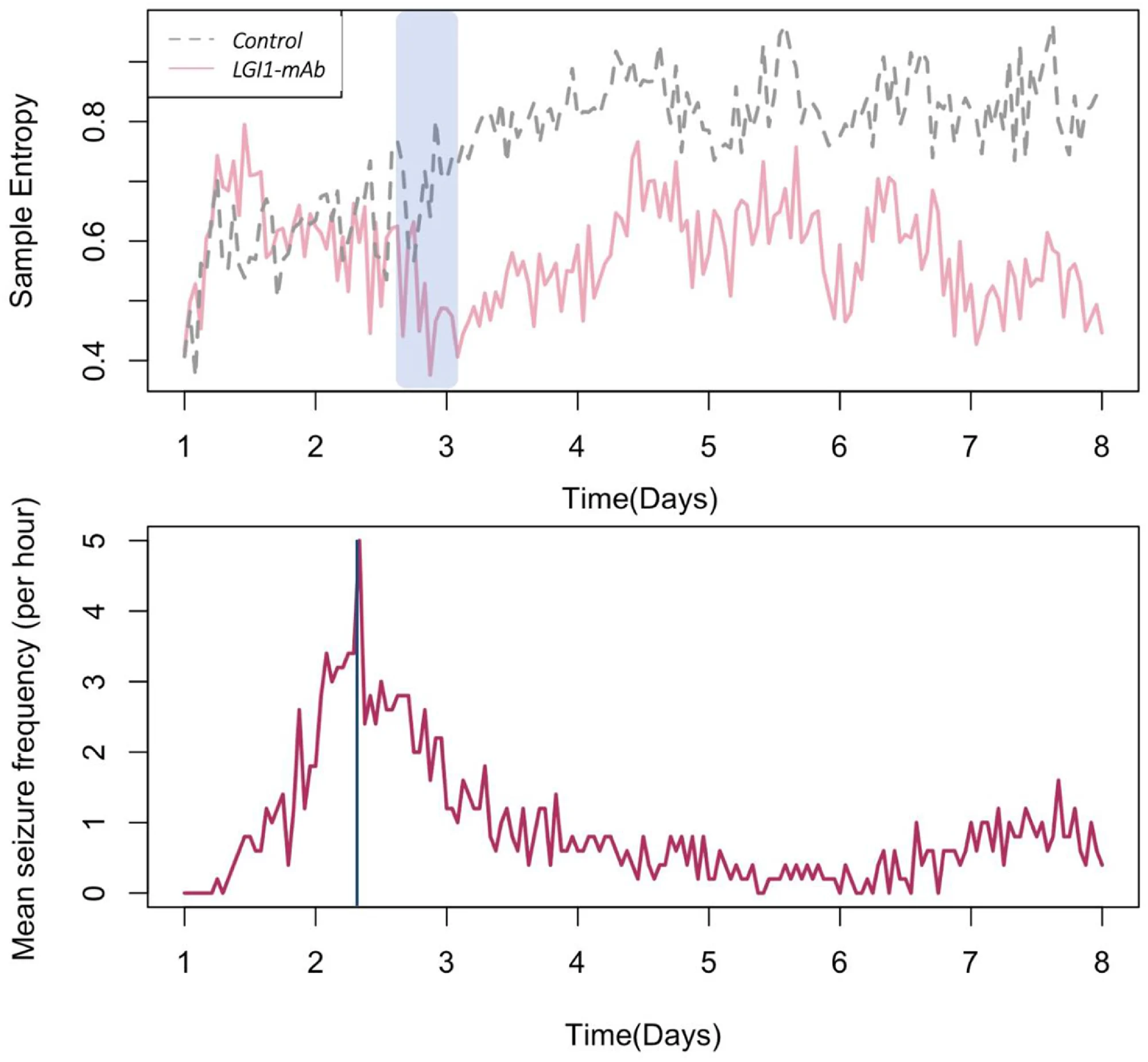
Time series plot of hourly mean SE/hour (A) and mean seizure frequency/hour in LGI1-mAb rats (B). (A) Time series graph depicting the mean SE per hour in LGI1-mAb infused rats (pink) and control rats (grey) (B) Time series graph of mean seizure frequency per hour in LGI1-mAb treated rats. We can see from the graph that a peak in seizure frequency between day 2 and 3 (blue solid line) is followed by a decrease in SE (blue shaded region) in the LGI1-mAb infused rats.

Lastly, we used a CCF function to test if there was a *lagged* correlation between seizure frequency and SE. Figure 5 shows the lagged correlation plot between the two time series. CCF values tending to 1 or −1 demonstrate a strong positive or negative correlation between the two time series respectively, while values closer to 0 indicate a lack of correlation between the two variables. The plot indicates that there is a negative correlation between seizure frequency and SE, and this correlation is most strongly observed between lags of approximately of 4–22 h. This is in line with the findings of figure 4, wherein a peak in mean seizure frequency between day two and three (∼5 seizures/hour, 33 h after treatment), is followed by the lowest observed value of mean SE (0.38) 13 h later.

**Figure 5.**
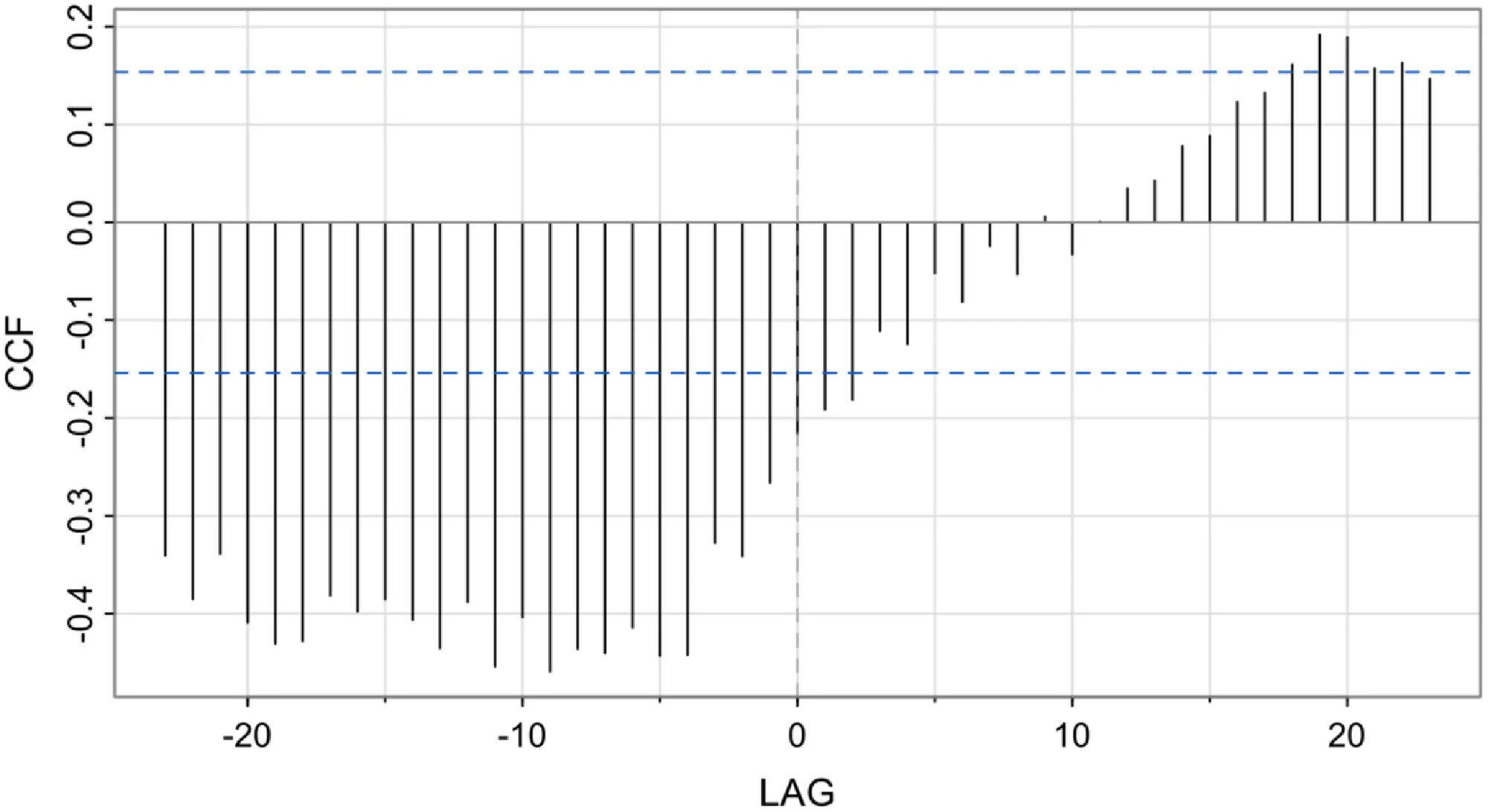
Lagged (cross-correlation) between mean seizure frequency/hour and mean sample entropy/hour. The *x*-axis shows the lag in hours between the two time series- (i) mean seizure frequency per hour and (ii) averaged SE per hour- in LGI1-mAb infused rodents (*n* = 5). The *y*-axis shows the CCF between the two series at each lag. The dominant negative-lag relationship in this plot indicates that SE ‘lags’ behind seizure frequency, or said another way- higher seizure frequency was associated with lower SE after a lag several hours. The strongest negative correlations (*r* < −0.4) was observed across lags of approximately 4–22 h between seizure frequency and SE time series.

## 5. Discussion

In this study we explored the relationship between the onset of seizures and the levels of entropy in the resting state cortex of humans and rodents. While human MEG provides a spatially rich insight into the association between the history of ictal events and entropy at a single point in time, EEG data from the LGI1-mAb infused rodent model provided exploratory temporal information between the association of seizures with entropy.

In disagreement with our primary hypothesis, we did not observe any significant difference between the epileptogenic and non-epileptogenic regions of the brain with respect to SE. On the contrary, we found that SE showed little variability between different regions of the same cortex. These results are in contrast to a recent study which found that resection of interictal ‘low entropy zones’ derived from intracranial recordings was predictive of better Engel outcomes (degree of seizure remission post-surgery) [54]. Another recent intraoperative ECoG study in patients with focal cortical dysplasia reported that higher post-resection spectral entropy variability in the fast-ripple band (250–500 Hz) was associated with poorer surgical outcome [55].

In the human MEG recordings, a backward stepwise regression model showed that an earlier age of seizure onset was associated with cortex wide lower entropy and greater variability of the same from one region to another. Parietal-lobe resection and barbiturate use were also associated with lower SE; however, the small number of patients in each subgroup means that these findings require validation in a larger cohort. Other clinical and demographic characteristics like seizure frequency, age at MEG scan and sex did not significantly explain the variance in the entropy of patients. These results are consistent with the hypothesis that epilepsy is a ‘network’ disorder, rather than a disorder confined to a single region of the brain [56]. In line with these results, we found sustained lower SE trajectory in the rodent model of LGI1 autoimmune encephalitis as compared to control rodents. Moreover, this sustained lower trajectory in LGI1-mAb rats persisted even after the initial peak of seizure frequency had passed.

Entropy falls drastically during an ictal event because neural activity during seizures becomes more synchronous and predictable [23–25]. This property of ictal discharges has been used extensively to detect and predict seizures from neurophysiological recordings [57, 58]. Previously, Urigüen *et al* [59] analysed background scalp EEG recordings in persons with idiopathic generalized epilepsy. They found that Shannon spectral entropy (SSE) tends to be *higher* in persons with idiopathic epilepsy as compared to healthy controls. However, they also found that SSE *decreases* with the advent of a seizure and approaches values close to those of healthy controls over a span of time, which may last up to 8 years. Our results are in partial agreement with Urigüen *et al* [59], since we observed sustained entropy in rodent models after seizure onset compared to controls.

### 5.1. Implications for early life epilepsy

It is well established that an earlier age of seizure onset is predictive of greater risk of developing drug resistance, mortality [60, 61] and is also associated with poor neurodevelopmental outcomes [62] and intellectual disability [63]. A pivotal study by Saxe *et al* calculated the brain entropy of resting state fMRI signals from a very large cohort of healthy individuals and found that higher entropy was associated with higher intelligence. They concluded that complex cognitive behaviour is strongly linked with access to varied neural states, and this information can be derived from the resting state signal alone [21]. Therefore, altered brain entropy offers a possible explanation for the association between early life epilepsies and poor intellectual development. Indeed, we observed that early onset was associated with lower brain entropy of the whole cortex even later in life.

Given the retrospective nature of this study, we were unable to retrieve healthy control data for human MEG recordings, especially since a large fraction of the study cohort was paediatric, and therefore not apt for comparison with healthy adult subjects. However, comparison of SE in individuals with epilepsy and healthy subjects can be a topic of future research in this area. As the cohort was restricted to patients who subsequently achieved Engel I outcomes, the generalizability of these findings to non-surgical patients, multifocal epilepsy, and patients with poorer surgical outcomes remains to be established. Also, we were unable to retrieve the IQ and pre-surgical neurocognitive outcomes for the patients in our cohort. As discussed above, early life epilepsy is associated with poorer cognitive outcomes, which may have confounded the association between age of seizure onset and cortical entropy. Another limitation of the study was that IEDs were not systematically quantified or removed from the resting-state MEG recordings. As IEDs form a part of the interictal neurophysiology of epilepsy, the present analysis therefore characterized them as a naturally occurring interictal signal. However, the present analysis cannot distinguish the contribution of IEDs from more sustained alterations in background cortical dynamics.

A limitation of the rodent experimental model used in our study is that Sleep–wake states were not explicitly controlled in the rodent entropy analysis; although LGI1-mAb and control animals were compared across time-matched hourly windows under the same light–dark cycle, differences in vigilance-state composition may have contributed to the observed entropy differences. Further, postictal intervals were not removed from the data, therefore these may contain periods of postictal suppression, potentially confounding sustained interictal alteration of entropy in LGI1-mAb rodents. Another limitation of the experimental model is that direct antibody-mediated effects on neuronal and network function cannot be fully disentangled from seizure-related effects on entropy.

## Data Availability

The data cannot be made publicly available upon publication because the cost of preparing, depositing and hosting the data would be prohibitive within the terms of this research project. The data that support the findings of this study are available upon reasonable request from the authors.
